# miR-145 improves metabolic inflammatory disease through multiple pathways

**DOI:** 10.1093/jmcb/mjz015

**Published:** 2019-04-03

**Authors:** Min He, Nan Wu, Man Cheong Leong, Weiwei Zhang, Zi Ye, Rumei Li, Jinyang Huang, Zhaoyun Zhang, Lianxi Li, Xiao Yao, Wenbai Zhou, Naijia Liu, Zhihong Yang, Xuehong Dong, Yintao Li, Lili Chen, Qin Li, Xuanchun Wang, Jie Wen, Xiaolong Zhao, Bin Lu, Yehong Yang, Qinghua Wang, Renming Hu

**Affiliations:** 1 Department of Endocrinology and Metabolism, Huashan Hospital, Shanghai Medical College, Institute of Endocrinology and Diabetology, Fudan University, Shanghai, China; 2 Department of Geriatrics, Zhongshan Hospital, Shanghai Medical College, Fudan University, Shanghai, China; 3 Division of Endocrinology and Metabolism, the Keenan Research Centre in the Li Ka Shing Knowledge Institute, St. Michael’s Hospital, University of Toronto, Ontario, Canada

**Keywords:** type 2 diabetes, osteoprotegerin, Kruppel-like factor 5, monocyte, NF-κB

## Abstract

Chronic inflammation plays a pivotal role in insulin resistance and type 2 diabetes, yet the mechanisms are not completely understood. Here, we demonstrated that serum LPS levels were significantly higher in newly diagnosed diabetic patients than in normal control. miR-145 level in peripheral blood mononuclear cells decreased in type 2 diabetics. LPS repressed the transcription of miR-143/145 cluster and decreased miR-145 levels. Attenuation of miR-145 activity by anti-miR-145 triggered liver inflammation and increased serum chemokines in C57BL/6 J mice. Conversely, lentivirus-mediated miR-145 overexpression inhibited macrophage infiltration, reduced body weight, and improved glucose metabolism in db/db mice. And miR-145 overexpression markedly reduced plaque size in the aorta in ApoE^−/−^ mice. Both OPG and KLF5 were targets of miR-145. miR-145 repressed cell proliferation and induced apoptosis partially by targeting OPG and KLF5. miR-145 also suppressed NF-κB activation by targeting OPG and KLF5. Our findings provide an association of the environment with the progress of metabolic disorders. Increasing miR-145 may be a new potential therapeutic strategy in preventing and treating metabolic diseases such as type 2 diabetes and atherosclerosis.

## Introduction

The prevalence of obesity and diabetes is increasing dramatically worldwide, while the underlying mechanism is not fully understood (Mokdad et al., 2001). Growing studies suggested that inflammation plays an important role in the development of obesity and diabetes. Inflammation occurring in adipose tissue has a broad-ranging impact on glucose, lipids, and energy metabolism (Hotamisligil et al., 1993; Sethi and Hotamisligil, 1999; Takahashi et al., 2003; Weisberg et al., 2003; Xu et al., 2003; Romeo et al., 2012). Obesity gives rise to a state of chronic, low-grade inflammation that contributes to insulin resistance and type-2 diabetes mellitus (T2DM) (Shoelson et al., 2007). Macrophage activation is one of the key signaling pathways involved in the pathogenesis of obesity-associated inflammation (Cinti et al., 2005; Strissel et al., 2007). Recent studies suggested that macrophage infiltration and activation in the adipose tissue provide a link between adipose tissue and inflammation, which in turn, lead to insulin resistance (Saltiel and Kahn, 2001; White, 2002; Weisberg et al., 2003; Xu et al., 2003). We proposed that diseases, including obesity, diabetes, fatty liver disease, atherosclerosis, are different phenotypes of metabolic inflammation induced by activated macrophage, which are collectively referred to as ‘metabolic inflammatory syndrome’ (MIS) (Hu et al., 2018).

MicroRNAs (miRNAs) comprise a broad class of small non-coding RNAs, that regulate genes at the posttranscriptional level through complementary base pairing to messenger RNAs (Bartel, 2004; Sun and Lai, 2013). Dysregulation of miRNAs has been found in various diseases (He et al., 2007; Mi et al., 2007), including metabolic diseases (Krutzfeldt and Stoffel, 2006; Trajkovski et al., 2011).

Kruppel-like factor 5 (KLF5), a member of the Kruppel-like factor family, is a transcription factor which regulates gene expressions through binding to GC box of gene promoter. KLF5 is widely expressed in mammals and is found to regulate genes involved in regulation of cell cycle, apoptosis, migration, and differentiation. Previous studies showed that KLF5 is an important mediator for stimuli-induced proinflammatory response through activation of NF-κB (i.e. subunits p50 and p65) (Chanchevalap et al., 2006; Baker et al., 2011).

Osteoprotegerin (OPG), a soluble member of the tumor necrosis factor receptor superfamily, is linked to cardiovascular disease and atherogenesis (Simonet et al., 1997; Schoppet et al., 2004; Tousoulis et al., 2013). OPG promotes the recruitment and infiltration of monocytes/macrophages and enhances monocyte locomotion *in vitro* (Moran et al., 2005; Mosheimer et al., 2005). Recent studies demonstrated that plasma OPG concentration is a strong and independent predictor of cardiovascular conditions, especially in patients with T2DM (Avignon et al., 2005, 2007; Nabipour et al., 2010).

In this study, we demonstrated that miR-145 levels were significantly lower in peripheral blood mononuclear cells of patients with T2DM compared with normal controls. Silencing miR-145 by a specific antisense oligonucleotide *in vivo* leads to increased infiltration of monocytes in the liver and an increase in serum pro-inflammatory factor. Mice treated with miR-145 lentivirus showed decreased body weight, improved glucose metabolism and ameliorated macrophage infiltration and atherosclerosis. OPG and KLF5 were proved to be the targets of miR-145 in ameliorating metabolic inflammation. These data suggested that miR-145 plays a critical role in the development of metabolic diseases and could be a potential candidate for treatment.

## Results

### miR-145 is downregulated in monocytes of patients with type 2 diabetes

**Figure 1:**
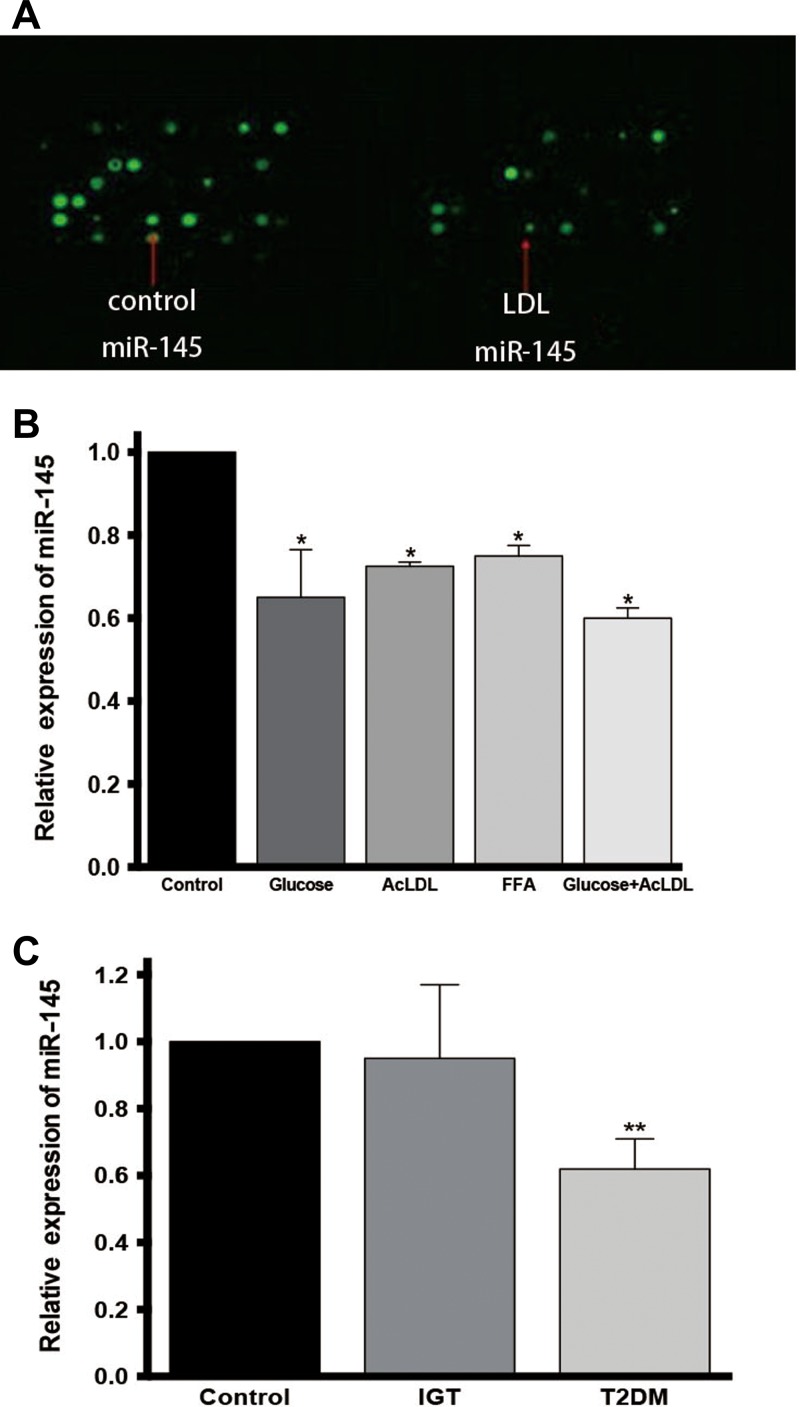
miR-145 is downregulated in high glucose and lipid-treated THP-1 cells and peripheral blood mononuclear cells in patients with type 2 diabetes. (**A**) miRNA microarray of LDL-treated THP-1 cells and controls. (**B**) qPCR of miR-145 in THP-1 cells treated with high glucose, Ac-LDL, FFA, Ac-LDL plus high glucose, and controls. (**C**) qPCR of miR-145 in peripheral blood mononuclear cells of patients newly diagnosed type 2 diabetes, with IGT, and control subjects.

A significant downregulation of miR-145 was found in THP-1 cells treated with high glucose, AcLDL and FFA by microRNA microarrays and was proved by quantitative Real-time polymerase chain reaction (qPCR) (Figure 1A and B; Supplementary Table S1). miR-145 levels in peripheral blood mononuclear cells was further compared among newly diagnosed T2DM patients, patients with impaired glucose tolerance (IGT), as well as normal subjects, and a significant decrease was found in T2DM patients compared with the other two groups (Figure 1C).

### Inhibition of miR-145 elicites inflammation in vivo

**Figure 2:**
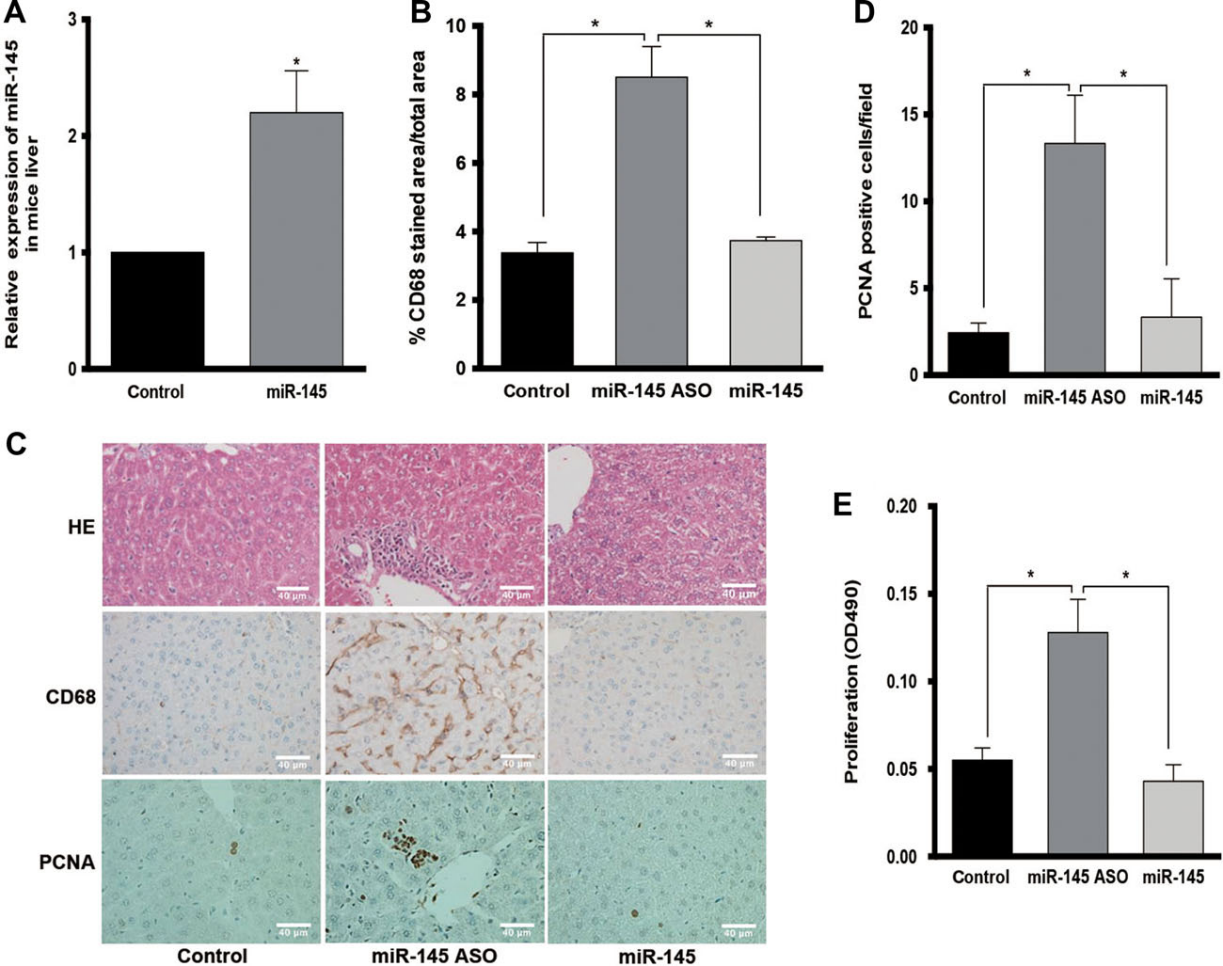
miR-145 induces macrophage proliferation and infiltration. (**A**) The level of miR-145 in C57BL/6 J mouse liver detected by qPCR after three days of oligonucleotide injection. (**B**) Quantification of CD68-positive areas (CD68-stained surface/total surface) in liver section. (**C**) Hematoxylin-and-eosin (HE) staining and IHC staining for CD68 and PCNA of the liver section from PBS, miR-145 ASO, or miR-145 oligonucleotide-treated C57BL/6 J mice. Original magnification, 400×. (**D**) Quantification of PCNA-positive cells in liver section from PBS, miR-145 ASO, or miR-145 oligonucleotide-treated C57BL/6 J mice. (**E**) Proliferation of BMMNCs treated with miR-145 or miR-145 ASO, detected using CellTiter 96 AQueous One Solution Cell Proliferation Assay.

To investigate the role of miR-145 in inflammation, miR-145 and miR-145 antisense oligonucleotide (ASO) were chemically synthesized and injected intravenously at a dose of 16 mg/kg to C57BL/6 J mice daily for 3 days, with PBS-treated group as control. Over 2-fold increase of miR-145 levels was observed in the liver of the mice treated with miR-145 oligonucleotide (Figure 2A). Immunohistochemistry showed more CD68-positive cells in the liver of miR-145 ASO-treated mice, which suggested more macrophage infiltration (Figure 2B and C). Immunohistochemical staining of proliferating cell nuclear antigen (PCNA), a cell proliferation marker, was found to be higher in the liver section of the miR-145 ASO group. Meanwhile a tendency for an increased number of PCNA-positive cells was noted in macrophage-rich regions of livers. (Figure 2C and D). These data suggested that inhibition of miR-145 stimulated macrophage infiltration and proliferation. Since macrophages are mainly derived from bone marrow precursor cells, bone marrow mononuclear cells (BMMNCs) were isolated and transfected with miR-145 or miR-145 ASO. The proliferation assay was performed and showed more active proliferation in BMMNCs transfected with miR-145 ASO (Figure 2E). The abundance of proinflammatory cytokines were also determined in the mice liver, and TNF-α and MCP-1 showed an increase in the miR-145 ASO group (Supplementary Table S2).

### miR-145 treatment improves glucose metabolism, reduces macrophage infiltration, and suppresses NF-κB activation in the liver in db/db mice

**Figure 3:**
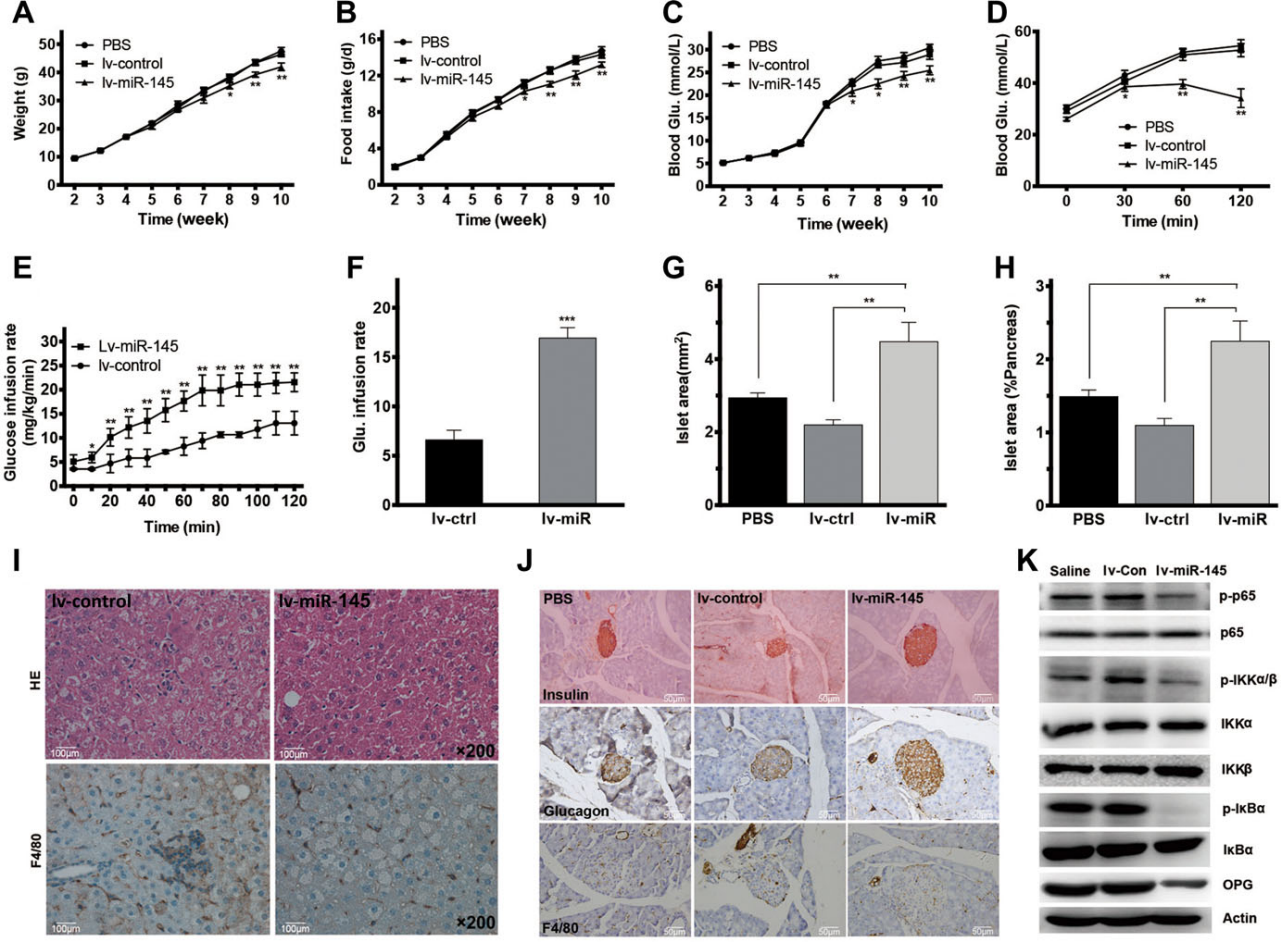
miR-145 overexpression improves metabolic disorders in db/db mice. (**A**) Weight curve of db/db mice treated with lv-miR-145, lv-control, or PBS. (**B**) Daily food intake curve of db/db mice treated with lv-miR-145, lv-control, or PBS. (**C**) Fasting blood glucose curve of db/db mice treated with lv-miR-145, lv-control, or PBS. (**D**) Glucose tolerance test after lv-miR-145, lv-control, or PBS treatment. (**E**) Glucose infusion rate at time 0–120 min during hyperinsulinaemic–euglycaemic clamp of db/db mice treated with lv-miR-145 or lv-control (*n* = 3 per group). (**F**) Steady-state glucose infusion rate during clamp (0–120 min). (**G**) Quantification of islet area detected by IHC (*n* = 3 mice per group). (**H**) Quantification of islet area expressed as the percentage of total pancreas area (*n* = 3 mice per group). (**I**) HE staining and IHC staining for F4/80 of the liver section from db/db mice treated with lv-miR-145 or lv-control. Original magnification, 200×. (**J**) HE and IHC staining for insulin, glucagon, and F4/80 of the pancreas section from db/db mice treated with lv-miR-145 or lv-control. Original magnification, 200×. (**K**) Western blot for NF-κB pathway molecules in the liver of lv-miR-145, lv-control, or saline-treated db/db mice.

To further address the role of miR-145 in metabolism and the underlying mechanism, lentiviral vector expressing miR-145 (lv-miR-145) was developed for stable miRNA expression. Two-week-old db/db mice were treated with lv-miR-145 and controls (lv-control and PBS). We noticed that mice treated with lv-miR-145 showed decreased weight, food intake, fasting blood glucose levels, as well as improved glucose tolerance (Figure 3A–D). Clamp experiment further showed a higher glucose infusion rate in the miR-145-treated group, which suggested improved insulin sensitivity (Figure 3E and F). Macrophage infiltration in the liver was observed using immunohistochemical staining of F4/80 and miR-145-treated group showed attenuated macrophage infiltration (Figure 3I). The mRNA expression of MCP-1, TNF-α, IL-1β, and IL-6 in the live was significantly reduced in the lv-miR-145-treated group (Supplementary Figure S1A and B). Immunohistochemical staining of F4/80 also found that miR-145 treatment reduced macrophage infiltration in the islets (Figure 3J), and β-cell area was increased in the lv-miR-145-treated group (Figure 3G and H). As a key nuclear transcriptional factor involved in inflammatory response and insulin resistance, NF-κB activation, as well as osteoprotegerin (OPG), was examined by western blot in the mouse liver sample to investigate the mechanism of miR-145 alleviating inflammation. The results showed decreased phosphorylated IκBα and phosphorylated p65 in miR-145-treated group, which suggested attenuated NF-κB activation. And OPG was also found decreased in the liver of miR-145-treated mice (Figure 3K). These findings indicated that overexpression of miR-145 decreased inflammatory reaction by inhibiting NF-κB activation in the liver.

To investigate the effect of miR-145 on inflammation in adipose tissue, we tested the expression of miR-145 in the omental adipose tissue of obese patients, diabetics, and normal subjects. miR-145 was found attenuated in obese, diabetic patients, while TNF-α and IL-6 were increased (Li et al., 2018).

### miR-145 alleviates atherosclerosis in ApoE^−/−^ mice

Accumulating evidences suggest that macrophage-mediated inflammation are involved in the development of atherogenesis. We have shown that miR-145 treatment reduced macrophage infiltration. In order to address whether miR-145 treatment can attenuate the development of vascular lesions, we performed early intervention studies in ApoE^−/−^ mice, a model of atherosclerosis on a western diet. Seven-week-old mice were treated with lv-miR-145 or controls and a western diet was started simultaneously. At 16 weeks of age, the mice were sacrificed. qPCR confirmed the overexpression of miR-145 in the aorta in lv-miR-145-treated group (Supplementary Figure S2A). Oil red staining and quantification of atherosclerosis of the *en face* aorta showed markedly reduced atherosclerotic lesion areas in the lv-miR-145-treated group (Supplementary Figure S2C and Table S3). Lv-miR-145 treatment also decreased the mean arterial blood pressure (Supplementary Figure S2B). However, the lipid metabolism in all the three groups was comparable (data not shown).

### Both OPG and KLF5 are targets of miR-145

**Figure 4:**
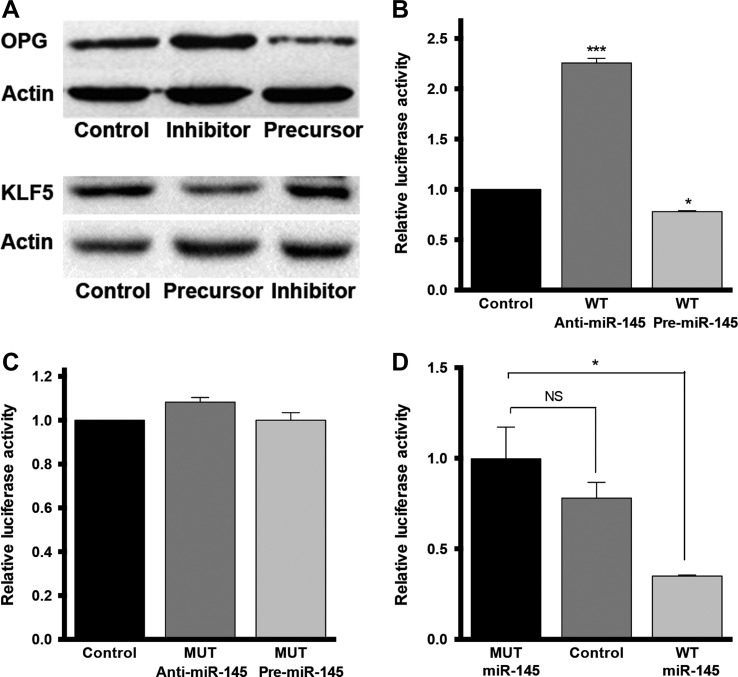
Both OPG and KLF5 are miR-145 target. (**A**) Western blot for OPG and KLF5 in THP-1 cells transfected with miR-145 inhibitor or precursor. (**B** and **C**) Dual luciferase reporter assay of miR-145 and 3’-UTR of OPG. (**D**) Dual luciferase reporter assay of miR-145 and 3’-UTR of KLF5.

miRNAs are negative regulators of gene expression. Using a bioinformatic approach, we identified OPG and Krueppel-like factor 5 (KLF5) as candidate targets of miR-145 (by TargetScan and MiRanda). THP-1 cells were transfected with miR-145 inhibitor or precursor. Western blot confirmed the negative regulation of miR-145 on the protein levels of both OPG and KLF5 (Figure 4A). Dual Luciferase reporter assay confirmed the direct interaction of miR-145 with the 3’-untranslated region (UTR) of OPG (Figure 4B and C) and KLF5 (Figure 4D).

### miR-145 represses monocyte proliferation partially by targeting OPG in vitro

**Figure 5:**
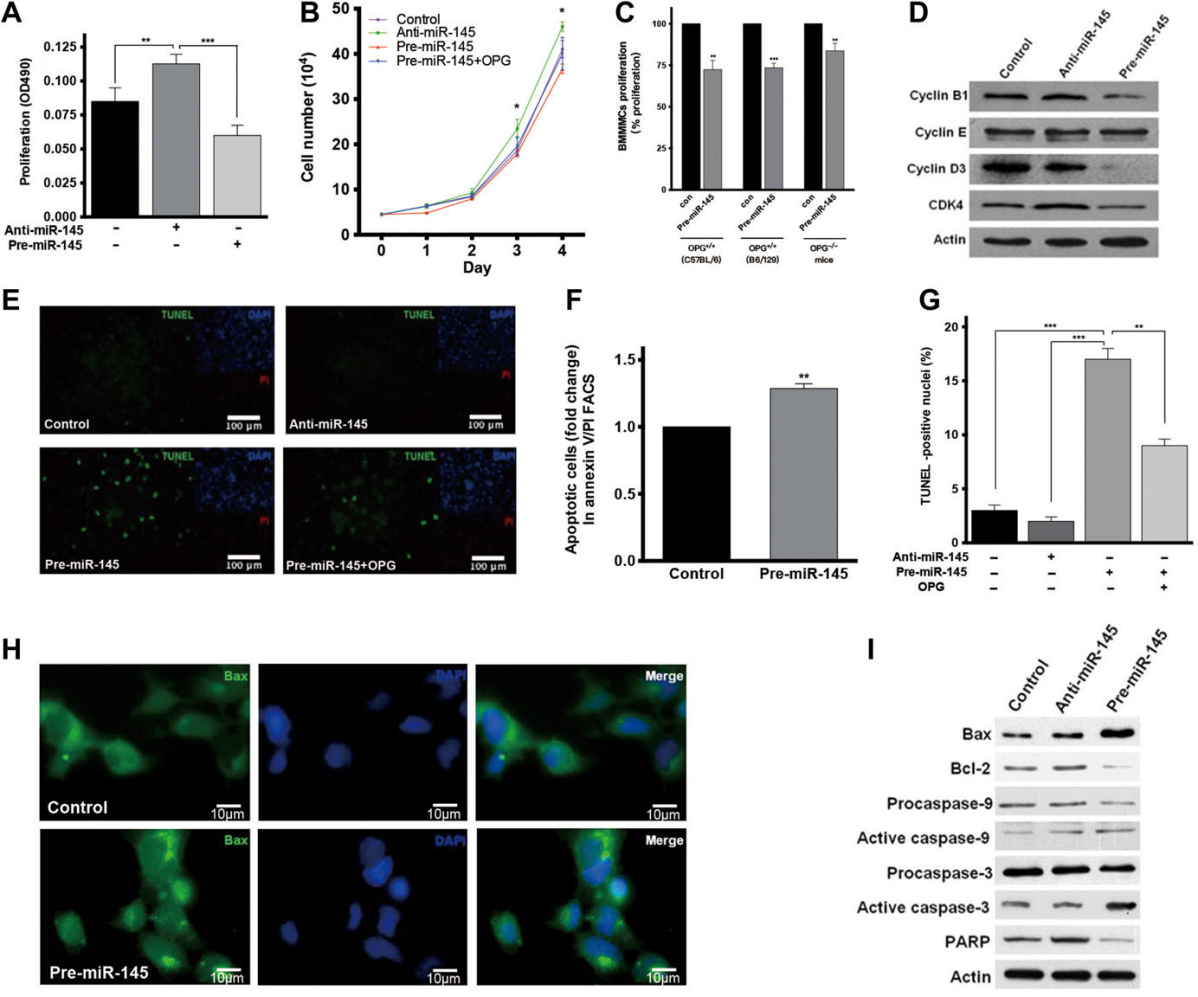
miR-145 represses cell proliferation and promotes apoptosis in THP-1 cells, BMMNCs, and HEK293 cells. (**A**) XTT assay at 72 h after transfection with anti-miR-145 or pre-miR-145 in THP-1 cells. (**B**) Cell proliferation curve of HEK293 cells after transfection with anti-miR-145 or pre-miR-145 with or without OPG. (**C**) Cell proliferation of BMMNCs isolated from wild-type or OPG^−/−^ mice at 72 h after transfection with pre-miR-145 or control. Cell proliferation is presented as the percentage relative to the proliferation rate of control. (**D**) Western blot for cyclin B1, cyclin E, cyclin D3, and CDK4 at 48 h after transfection with anti-miR-145 or pre-miR-145 in HEK293 cells. (**E**) TUNEL assay of HEK293 cells transfected with anti-miR-145 or pre-miR-145 with or without OPG (10 ng/ml). (**F**) Apoptosis assay using annexin V/PI FACS in HEK293 cells transfected with pre-miR-145. (**G**)The proportion of apoptotic cells in **E**. (**H**) Bax distribution in HEK293 cells transfected with pre-miR-145, detected by immunofluorescence staining. (**I**) Western blot for apoptotic-related proteins in HEK293 cells transfected with pre-miR-145 or anti-miR-145.

As we have demonstrated that BMMNCs transfected with miR-145 ASO showed more active proliferation, we further investigated the effect of miR-145 on cell proliferation in THP-1 and HEK293 cells to confirm such effect. THP-1 cells were treated with pre-miR-145 or anti-miR-145. XTT assay showed that inhibition of miR-145 promoted cell proliferation, while miR-145 treatment suppressed cell proliferation (Figure 5A). Cell proliferation curve of HEK293 cells also confirmed the proliferation repression effect of miR-145 (Figure 5B). Since OPG was reported to be associated with cell apoptosis through a p53-independent mechanism (Ashkenazi, 2002), we investigated the role of OPG in this process. The proliferation curve of HEK293 cells showed that OPG blocked the proliferation repression effect of miR-145 (Figure 5B). To confirm whether the repression effect of miR-145 is OPG-dependent or not, we further performed the proliferation assay using BMMNCs isolated from OPG knockout (OPG^−/−^) mice. We found that ectopic expression of miR-145 in OPG^−/−^ cells preserved about one third of the inhibition on cell proliferation (Figure 5C), suggesting that miR-145 repressed cell proliferation partially dependent on OPG. To investigate the mechanism of miR-145 repressing cell proliferation, the change of cell cycle regulators including cyclin B1, cyclin E, cyclin D3, and cyclin-dependent kinase 4 (CDK4) was examined in anti-miR-145 and pre-miR-145-treated cells. Cyclin B1, cyclin D3, and CDK4 were found decreased in miR-145-treated cells (Figure 5D).

### miR-145 induces monocyte apoptosis partially by targeting OPG in vitro

A similar strategy was employed to study the role of miR-145 and OPG in cell apoptosis. Terminal deoxynucleotidyl transferase-mediated deoxyuridine triphosphate nick-end labeled (TUNEL) assay and FACS showed that pre-miR-145 treatment significantly increased HEK293 cell apoptosis, while OPG treatment partially inhibited the effect (Figure 5E–G). To elucidate the mechanism of the pro-apoptosis effect of miR-145, we examined the pivotal mediators of intrinsic and extrinsic apoptosis pathways in HEK293 cells. We found that pre-miR-145 treatment increased punctuated distribution of Bax (Figure 5H). miR-145 increased the levels of Bax, active caspase-3, and active caspase-9 whereas decreased Bcl-2 and poly (ADP-ribose) polymerase (PARP) levels (Figure 5I). The pro-apoptosis effect of miR-145 still existed in p53-null HEK293 cells, which suggested that miR-145 may cause pro-apoptotic effects independent of p53 (data not shown).

### miR-145 suppresses LPS-induced NF-κB activation by KLF5 and OPG in vitro

**Figure 6:**
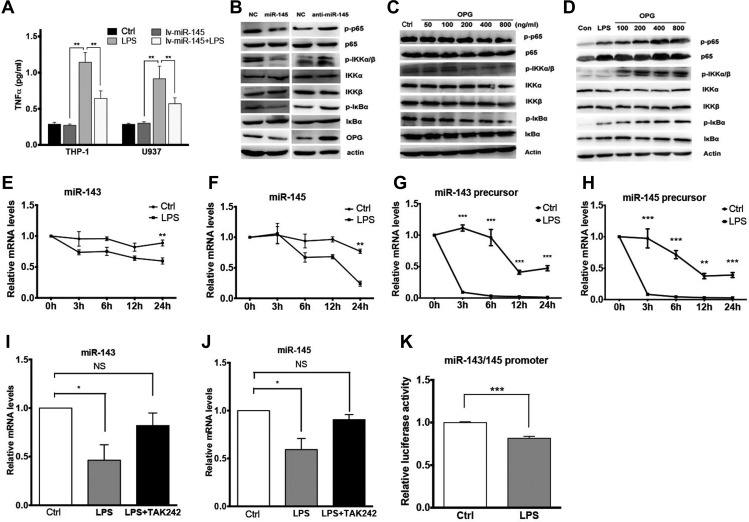
miR-145 suppresses NF-κB activation and LPS represses the transcription of miR-143/145 cluster. (**A**) TNF-α concentration in the supernatant of THP-1 or U937 cells transfected with lv-miR-145 or treated with LPS or both. (**B**) Western blot for NF-κB pathway molecules in THP-1 cells transfected with 100 nM miR-145 mimics, anti-miR-145, or negative control mimics (NC) and stimulated with LPS. (**C**) Western blot for NF-κB pathway molecules in THP-1 cells treated with OPG at different concentrations. (**D**) Western blot for NF-κB pathway molecules in THP-1 cells pre-treated with OPG at different concentrations and stimulated with LPS. (**E**) qPCR for miR-143 level in THP-1 cells stimulated with LPS (100 μg/ml) for different times. (**F**) qPCR for miR-145 level in THP-1 cells stimulated with LPS (100 μg/ml) for different times. (**G**) qPCR for miR-143 precursor level in THP-1 cells stimulated with LPS (100 μg/ml) for different times. (**H**) qPCR for miR-145 precursor level in THP-1 cells stimulated with LPS (100 μg/ml) for different times. (**I**) qPCR for miR-143 level in THP-1 cells after LPS stimulation with or without TAK242 (antagonist of TLR4). (**J**) qPCR for miR-145 level in THP-1 cells after LPS stimulation with or without TAK242. (**K**) Dual luciferase reporter assay of the promoter of miR-143/145 cluster with or without LPS stimulation.

Since miR-145 was downregulated in monocyte treated with high glucose, AcLDL, and FFA as well as in T2DM patients (Figure 1) and miR-145 alleviated inflammation and macrophage infiltration in C57BL/6 J, db/db, and ApoE^−/−^ mice (Figures 2 and 3), we tried to investigate the role of miR-145 in macrophage inflammatory response. THP-1 cells and U937 cells were transfected with lv-miR-145 or lv-miR-control. Transfected cells were then analyzed for LPS-induced TNF-α production, which is a key proinflammatory cytokine implicated in the development of insulin resistance, by ELISA assay. Cells treated with LPS showed a marked increase in TNF-α level, which was profoundly attenuated by miR-145 transduction. (Figure 6A). To investigate the effect of miR-145 on NF-κB activation *in vitro*, THP-1 cells were transfected with miR-145 mimics, antisense, and miR-NC and then stimulated with LPS. Western blot analysis showed that miR-145 decreased the phosphorylation of IκBα and p65 induced by LPS stimulation (Figure 6B).

The effects of KLF5 on promoting NF-κB activation and mediating inflammation have been reported (Chen et al., 2014; Ma et al., 2017; Zhang et al., 2017). Since the effect of OPG on macrophage inflammatory response was not clearly understood, here we examined the effects of OPG on NF-κB activation. Western blot analysis showed that the LPS-induced phosphorylation of p65, IKKα/β, and IκBα were increased in cells pretreated with OPG (Figure 6D). However, without LPS, OPG treatment decreased the phosphorylation of p65, IKKα/β, and IκBα (Figure 6C). This suggested the complexity of OPG effect on NF-κB activation, which needs further study. Nevertheless, the results suggested that miR-145 suppressed LPS-induced NF-κB activation by KLF5 and OPG *in vitro*.

*LPS decreases miR-145 through repressing the transcription of miR-143/145 cluster*

Next, we investigated the role of LPS in miR-145 regulation. Since miR-145 and miR-143 are clustered gene, levels of miR-143, miR-145, and their precursors in THP-1 cells before and after stimulation with LPS were all quantified by qPCR. The level of mature miR-145 was found decreased in 6 h and miR-143 was decreased in 3 h after LPS stimulation (Figure 6E and F). The precursors of miR-143 and miR-145 were significantly reduced 3 h after LPS stimulation (Figure 6G and H). TAK242, antagonist of toll-like receptor 4 (TLR4) could reverse the repression effect of LPS on miR-145 and miR-143 (Figure 6I and J). To further investigate the mechanisms of LPS decreasing the miR-143/145 cluster, we inspected the highly conserved miR-143/145 copromoter (610 bp upstream to 320 bp downstream of the TSS of miR-143HG) and dual luciferase reporter assay was conducted. The result showed that LPS repressed the transcription of miR-143/145 cluster (Figure 6K).

## Discussion

A principal mechanistic core of type 2 diabetes and atherosclerosis resides at the interface of the metabolic and inflammatory pathways (Shoelson et al., 2006). Data from the present study suggest that miR-145 in peripheral blood mononuclear cells of newly diagnosed diabetic patients was downregulated. miR-145-based therapy is associated with a marked decrease in inflammatory status, improved glucose metabolism in db/db mice, and reduction in atherosclerotic plaque size in ApoE^−/−^ mice. The current data underscores the potential role of miR-145 in progress of diabetes and its critical therapeutic potential in attenuating type-2 diabetes and atherosclerosis.

In order to understand whether miR-145 contributes to development of inflammation in diabetic patients, we used 2 different approaches: inhibition of miR-145 and overexpression of miR-145. Using *in vivo* 2’-O-methyl modified ASO targeting miR-145, we provided an unambiguous demonstration that inhibition of miR-145 induced macrophage infiltration in the liver and mononuclear cells proliferation in bone marrow, while overexpression of miR-145 attenuated that in C57BL/6 J mice. Notably, injection of lentivirus-carrying miR-145 significantly reduced macrophage infiltration in liver and pancreatic island in db/db mice. The data verified that miR-145 attenuated macrophage activation and the onset of inflammation.

Moreover, miR-145 has been shown to be abundantly expressed in the vessel wall (Cheng et al., 2009). In previous studies, miR-145 knockout mice showed a noticeably thinner smooth muscle layer of arteries, which lead to moderate systemic hypotension (Elia et al., 2009; Xin et al., 2009). Caruso et al. (2012) demonstrated that the reduction of miR-145 protected against the development of pulmonary artery hypertension (PAH) in mice by targeting SMAD4 and SMAD5. On the contrary, Lovren et al. (2012) have recently reported that miR-145-treated mice showed a 60% reduction in plaque size and reduced macrophage infiltration by targeting KLF4, providing support for our findings that miR-145 reduced atherosclerosis. These findings suggest the complexity of miR-145 in inflammation regulation. Nevertheless, in atherosclerosis models, miR-145 may play a protective role.

OPG was confirmed the target of miR-145 in our study. Growing evidences indicate that serum levels of OPG are significantly increased in both diabetic and non-diabetic patients susceptible to atherosclerosis or vascular dysfunction (Simonet et al., 1997; Schoppet et al., 2002, 2004; Tousoulis et al., 2013). The interaction of OPG/TRAIL is biologically important in vascular physiopathology. TRAIL ameliorates atherosclerosis initiation and progression by inducing apoptosis of macrophages and neutrophils, resulting in macrophage clearance from atherosclerotic plaques (Kaplan et al., 2000; Renshaw et al., 2003). By contrast, OPG promotes the recruitment and infiltration of monocytes/macrophages and enhances monocyte locomotion *in vitro* (Moran et al., 2005; Mosheimer et al., 2005). The current study has also revealed that elevated OPG levels promote cell proliferation, impede apoptosis and attenuate LPS induced activation of NF-κB. Although the role of OPG in normal and pathological vasculature has been elucidated, little is known about the regulation of its expression. Our observations provided the possible regulatory mechanism of OPG in metabolic disorders.

However, we could only partially explain the anti-inflammatory effect of miR-145 by targeting OPG. According to the nature of miRNAs, we speculated that miR-145 should regulate inflammatory response by targeting multiple proteins. KLF-5 was predicted to be another target of miR-145, which was confirmed in our study. LPS induced KLF5 and the effect was antagonized by miR-145 in macrophages. Previous studies have found that KLF5 can stimulate inflammation and activate the NF-κB pathway. Therefore, we hypothesized that miR-145 ameliorates inflammation partially by targeting KLF5.

Evidences are growing that dysbiosis of intestinal flora may drive the onset of metabolic diseases. As a result of dysbiosis of intestinal flora, LPS levels increase. Our findings showed that LPS repressed the transcription of miR-143/145 cluster, resulting in the decrease of miR-145, providing an association of environment with the progress of metabolic disorders.

Since miR-145 attenuated body weight, glucose metabolism, atherosclerosis and macrophage infiltration in liver, we speculated that four metabolic diseases including atherosclerosis, NAFLD, obesity, and type 2 diabetes may share common origin. We propose that metabolic inflammatory syndrome (MIS) is a continuation of the metabolic syndrome (MS) and patients may be diagnosed with MIS when they suffer from two or more diseases of the four metabolic diseases.

In conclusion, the current study gives a picture of how the environment drives the onset of metabolic diseases, and provides the first report of miR-145-based therapeutic approach in the treatment of experimental diabetes and atherosclerosis. Changes in lifestyle and high fat diet may cause dysbiosis of intestinal flora, thus increasing LPS levels and decreasing miR-145, driving metabolic inflammation and causing metabolic disorders such as diabetes and atherosclerosis. miR-145 treatment attenuated macrophage infiltration and inflammatory cytokine secretion. Stable and long-term miR-145 treatment effectively reduced body weight, improved glucose metabolism and attenuated atherosclerotic plaque formation. Hence, strategies to upregulate miR-145 may be a useful treatment strategy for metabolic disorders.

## Materials and methods

### Cells and reagents

THP-1 cells and U937 cells were grown in RPMI 1640 medium containing 10% fetal bovine serum (FBS) and maintained at 37°C and 5% CO_2_ atmosphere. For all experiments, THP-1 cells or U937 cells were cultured at a suitable density and treated with PMA (160 nM for THP-1 cells and 20 nM for U937 cells) for 24 h to induce macrophage activation. Human peripheral blood mononuclear cells were isolated from blood obtained from 17 normal subjects, 16 patients with newly diagnosed impaired glucose tolerance (IGT), and 17 patients with T2DM by Ficoll density gradient centrifugation. Mouse bone marrow mononuclear cells (BMMNCs) were isolated from tibiofibula by Ficoll density gradient centrifugation. Primary antibodies against F4/80 and CD68 and secondary antibodies were purchased from Abcam. Primary antibodies against cyclin B1, cyclin E, cyclin D3, CDK4, p53, caspase-9, caspase-3, cleaved caspase-3, and PARP were purchased from Cell Signaling Technology. Primary antibodies against Bcl-2 and Bax were from Upstate and Chemicon, respectively.

### miRNA microarray assay and verification

THP-1 cells were treated with 60 mg/L Ac-LDL, 15 mM glucose, 0.5 mM FFA, or Ac-LDL plus high glucose, respectively. RNA was isolated using mirVana miRNA Isolation Kit (Ambion) and miRCURY^TM^ Array microarray kit (Cat# 208000V7.1, Exiqon) was used to investigate the differentially expressed miRNAs according to the manufacturer’s instructions. Differentially expression of miR-145 was verified using the mirVana qRT-PCR miRNA detection kit and mirVana qRT-PCR Primer set (Ambion). U6 was used as an internal control (Ambion).

### Cell proliferation assays

THP-1 cells were transfected with pre-miR-145 or anti-miR-145 by electroporation. HEK293 cells were transfected with pre-miR-145 or anti-miR-145 by Lipofectamin 2000. BMMNCs were transfected with pre-miR-145 by electroporation. After transfection, THP-1 cells and BMMNCs were seeded at a density of 6 × 10^3^−10 × 10^3^ cells per well in 96-well plate. After 72 h, THP-1 cell proliferation was assessed using XTT assay according to the manufacturer’s instructions. BMMNCs proliferation was assessed by CellTiter96 Aqueous One Solution Cell Proliferation Assay (Promega). For HEK293 cells, cell numbers were counted at the indicated time points for growth curves.

### Cell apoptosis assays

Apoptosis was assessed in mouse BMMNCs or HEK293 cells treated with pre-miR-145 and anti-miR-145 by FACS analysis of annexin V/propidium labeling (BioVision). Western blot was performed to detect Bcl-2, Bax, p53, caspase-9, caspase-3, cleaved caspase-3, and PARP. TUNEL was performed with a TUNEL kit (Upstate) as described by the manufacturer. TUNEL-positive cells were detected with fluorescent microscopy.

### Plasmid construction and lentivirus packaging

The pri-mir-145 was amplified by PCR with mouse genomic DNA. The sequences of the primers were as follows: F: 5′-CGGAATTCAAGGTCACTAGAGCCTGGGAAC-3′; R: 5′-CGCGGATCCTTCAACCACTGTGTCTTGAGAC-3′.

The PCR products were digested by *Eco*RI and *Bam*HI, and a 606-bp fragment containing pri-mir-145 was cloned into the lentiviral vector pCDH-CMV-MCS-EF1-copGFP (System Biosciences, Cat#CD511A-1) and the sequences were verified by DNA sequencing, which was named pLV-mir-145. The 293TN Producer Cell Line (System Biosciences, Cat#LV900A-1) was maintained in D-MEM (Invitrogen) plus 10% FBS (Invitrogen). pLV-mir-145 or pLV GFP control vector and pPACK Packaging Plasmid Mix (SBI) were co-transfected into 293TN cells using Lipofectamine 2000 according to the manufacturer’s instructions. Forty-eight hours after transfection, the supernatant was harvested and centrifugation at 5000× *g* at 4°C for 5 min and then filtered through a 0.45-μm syringe filter. The viral titer was determined using gradient dilution. The packaged lentiviruses were named lv-miR-145 and lv-control.

### Mouse experiments

Ten-week-old male C57BL/6 J mice (purchased from SLACCAS, Shanghai, China) were anesthetized and catheterized via the jugular vein. After one week of recovery, the mice were randomized into three groups. miR-145 sense oligonucleotide and antisense oligonucleotide (miR-145 ASO) were dissolved in sterile PBS (4 mg/ml) and injected intravenously at a dose of 16 mg/kg daily for three days. The mice were then sacrificed for the subsequent experiments.

Two-week-old male db/db mice (purchased from Model Animal Research Center of Nanjing University, Nanjing, China) were randomized into three groups and were treated every other day with lv-miR-145, lv-control, or PBS, respectively. For each mouse, a total of five injections were given. Intraperitoneal injection was given for the first two times, and intravenous injection via the caudal vein was carried out for the rest. The dose of virus for each injection was 1 × 10^7^ ifu. Body weight, food intake, and fasting blood glucose were evaluated weekly, and a glucose tolerance test was performed by intraperitoneal glucose injection (1.5 g/kg) at 10 weeks of age.

Seven-week-old male *ApoE*^−/−^ mice in C57BL/6 J background (purchased from Model Animal Research Center of Nanjing University) were fed a high-cholesterol atherogenic western diet. The mice were randomized into three groups and treated as the same regimen db/db mice. Arterial pressure was measured weekly until 16 weeks of age when the mice were sacrificed.

### Hyperinsulinemic–euglycemic clamps

Hyperinsulinemic–euglycemic clamps were performed in overnight-fasted halothane-anesthetized mice. Mice were catheterized, 20% D-glucose and insulin were infused via the saphenous vein, and then blood sampling was conducted through the femoral artery. Insulin (Humulin-R; Eli Lilly) was infused at 7.24 U/kg/min, beginning at *t* = –60 min and continuing until *t* = +120 min. Glucose was infused at a variable rate to maintain euglycemia, determined by glucose sampling and analysis at 5-min intervals (glucose oxidase method, YSI 2300-Stat Analyzer). Mean plasma glucose was maintained at 6.2–7.2 mM over 0 to 120 min period (mean CV 5.0%). Glucose infusion rate (GIR) was recorded from *t* = 0 to *t* = 120 min.

### Islet quantification

Pancreas were fixed in 10% neutral formalin and paraffin-embedded, and sections were HE-stained. Three whole sections were photographed, and areas of all visible islets and the whole section were analyzed in the IMS cell imagine analysis system using immunohistochemical quantitative analysis software.

### Statistical analysis

GraphPad Prism version 6.0 was used for statistical analysis. Data differences between groups were analyzed using ANOVA test. Differences were considered statistically significant at *P* < 0.05.

## Supplementary Material

mjz015_Supplementary_material

## References

[mjz015C1] Ashkenazi A. (2002). Targeting death and decoy receptors of the tumour-necrosis factor superfamily. Nat. Rev. Cancer 2, 420–430. 12189384 10.1038/nrc821

[mjz015C2] Avignon A., Sultan A., Piot C., et al. (2005). Osteoprotegerin is associated with silent coronary artery disease in high-risk but asymptomatic type 2 diabetic patients. Diabetes Care 28, 2176–2180. 16123486 10.2337/diacare.28.9.2176

[mjz015C3] Avignon A., Sultan A., Piot C., et al. (2007). Osteoprotegerin: a novel independent marker for silent myocardial ischemia in asymptomatic diabetic patients. Diabetes Care 30, 2934–2939. 17712025 10.2337/dc07-0992

[mjz015C4] Baker R.G., Hayden M.S., and Ghosh S. (2011). NF-κB, inflammation, and metabolic disease. Cell Metab. 13, 11–22. 21195345 10.1016/j.cmet.2010.12.008 PMC3040418

[mjz015C5] Bartel D.P. (2004). MicroRNAs: genomics, biogenesis, mechanism, and function. Cell 116, 281–297. 14744438 10.1016/s0092-8674(04)00045-5

[mjz015C6] Caruso P., Dempsie Y., Stevens H.C., et al. (2012). A role for miR-145 in pulmonary arterial hypertension: evidence from mouse models and patient samples. Circ. Res. 111, 290–300. 22715469 10.1161/CIRCRESAHA.112.267591

[mjz015C7] Chanchevalap S., Nandan M.O., McConnell B.B., et al. (2006). Kruppel-like factor 5 is an important mediator for lipopolysaccharide-induced proinflammatory response in intestinal epithelial cells. Nucleic Acids Res. 34, 1216–1223. 16500892 10.1093/nar/gkl014 PMC1383625

[mjz015C8] Chen H.L., Chong I.W., Lee Y.C., et al. (2014). Kruppel-like factor 5 mediates proinflammatory cytokine expression in lipopolysaccharide-induced acute lung injury through upregulation of nuclear factor-κB phosphorylation in vitro and in vivo. Mediators Inflamm. 2014, 281984. 25197166 10.1155/2014/281984 PMC4146351

[mjz015C9] Cheng Y., Liu X., Yang J., et al. (2009). MicroRNA-145, a novel smooth muscle cell phenotypic marker and modulator, controls vascular neointimal lesion formation. Circ. Res. 105, 158–166. 19542014 10.1161/CIRCRESAHA.109.197517 PMC2728297

[mjz015C10] Cinti S., Mitchell G., Barbatelli G., et al. (2005). Adipocyte death defines macrophage localization and function in adipose tissue of obese mice and humans. J. Lipid Res. 46, 2347–2355. 16150820 10.1194/jlr.M500294-JLR200

[mjz015C11] Elia L., Quintavalle M., Zhang J., et al. (2009). The knockout of miR-143 and -145 alters smooth muscle cell maintenance and vascular homeostasis in mice: correlates with human disease. Cell Death Differ. 16, 1590–1598. 19816508 10.1038/cdd.2009.153 PMC3014107

[mjz015C12] He L., He X., Lim L.P., et al. (2007). A microRNA component of the p53 tumour suppressor network. Nature 447, 1130–1134. 17554337 10.1038/nature05939 PMC4590999

[mjz015C13] Hotamisligil G.S., Shargill N.S., and Spiegelman B.M. (1993). Adipose expression of tumor necrosis factor-α: direct role in obesity-linked insulin resistance. Science 259, 87–91. 7678183 10.1126/science.7678183

[mjz015C14] Hu R., Xie Y., Lu B., et al. (2018). Metabolic inflammatory syndrome: a novel concept of holistic integrative medicine for management of metabolic diseases. AME Med. J. 51, 1–8.

[mjz015C15] Kaplan M.J., Ray D., Mo R.R., et al. (2000). TRAIL (Apo2 ligand) and TWEAK (Apo3 ligand) mediate CD4^+^ T cell killing of antigen-presenting macrophages. J. Immunol. 164, 2897–2904. 10706675 10.4049/jimmunol.164.6.2897

[mjz015C16] Krutzfeldt J., and Stoffel M. (2006). MicroRNAs: a new class of regulatory genes affecting metabolism. Cell Metab. 4, 9–12. 16814728 10.1016/j.cmet.2006.05.009

[mjz015C17] Li R., Shen Q., Wu N., et al. (2018). MiR-145 improves macrophage-mediated inflammation through targeting Arf6. Endocrine 60, 73–82. 29388044 10.1007/s12020-018-1521-8

[mjz015C43] Lovren F., Pan Y., Quan A., et al. (2012). MicroRNA-145 targeted therapy reduces atherosclerosis. Circulation 126, S81–S90. 22965997 10.1161/CIRCULATIONAHA.111.084186

[mjz015C18] Ma D., Zhang R.N., Wen Y., et al. (2017). 1, 25(OH)2D3-induced interaction of vitamin D receptor with p50 subunit of NF-κB suppresses the interaction between KLF5 and p50, contributing to inhibition of LPS-induced macrophage proliferation. Biochem. Biophys. Res. Commun. 482, 366–374. 27856242 10.1016/j.bbrc.2016.11.069

[mjz015C19] Mi S., Lu J., Sun M., et al. (2007). MicroRNA expression signatures accurately discriminate acute lymphoblastic leukemia from acute myeloid leukemia. Proc. Natl Acad. Sci. USA 104, 19971–19976. 18056805 10.1073/pnas.0709313104 PMC2148407

[mjz015C20] Mokdad A.H., Bowman B.A., Ford E.S., et al. (2001). The continuing epidemics of obesity and diabetes in the United States. JAMA 286, 1195–1200. 11559264 10.1001/jama.286.10.1195

[mjz015C21] Moran C.S., McCann M., Karan M., et al. (2005). Association of osteoprotegerin with human abdominal aortic aneurysm progression. Circulation 111, 3119–3125. 15939823 10.1161/CIRCULATIONAHA.104.464727

[mjz015C22] Mosheimer B.A., Kaneider N.C., Feistritzer C., et al. (2005). Syndecan-1 is involved in osteoprotegerin-induced chemotaxis in human peripheral blood monocytes. J. Clin. Endocrinol. Metab. 90, 2964–2971. 15728209 10.1210/jc.2004-1895

[mjz015C23] Nabipour I., Kalantarhormozi M., Larijani B., et al. (2010). Osteoprotegerin in relation to type 2 diabetes mellitus and the metabolic syndrome in postmenopausal women. Metabolism 59, 742–747. 19922962 10.1016/j.metabol.2009.09.019

[mjz015C24] Renshaw S.A., Parmar J.S., Singleton V., et al. (2003). Acceleration of human neutrophil apoptosis by TRAIL. J. Immunol. 170, 1027–1033. 12517970 10.4049/jimmunol.170.2.1027

[mjz015C25] Romeo G.R., Lee J., and Shoelson S.E. (2012). Metabolic syndrome, insulin resistance, and roles of inflammation--mechanisms and therapeutic targets. Arterioscler. Thromb. Vasc. Biol. 32, 1771–1776. 22815343 10.1161/ATVBAHA.111.241869 PMC4784686

[mjz015C26] Saltiel A.R., and Kahn C.R. (2001). Insulin signalling and the regulation of glucose and lipid metabolism. Nature 414, 799–806. 11742412 10.1038/414799a

[mjz015C27] Schoppet M., Al-Fakhri N., Franke F.E., et al. (2004). Localization of osteoprotegerin, tumor necrosis factor-related apoptosis-inducing ligand, and receptor activator of nuclear factor-κB ligand in Monckeberg’s sclerosis and atherosclerosis. J. Clin. Endocrinol. Metab. 89, 4104–4112. 15292354 10.1210/jc.2003-031432

[mjz015C28] Schoppet M., Preissner K.T., and Hofbauer L.C. (2002). RANK ligand and osteoprotegerin: paracrine regulators of bone metabolism and vascular function. Arterioscler. Thromb. Vasc. Biol. 22, 549–553. 11950689 10.1161/01.atv.0000012303.37971.da

[mjz015C29] Sethi J.K., and Hotamisligil G.S. (1999). The role of TNFα in adipocyte metabolism. Semin. Cell Dev. Biol. 10, 19–29. 10355025 10.1006/scdb.1998.0273

[mjz015C30] Shoelson S.E., Herrero L., and Naaz A. (2007). Obesity, inflammation, and insulin resistance. Gastroenterology 132, 2169–2180. 17498510 10.1053/j.gastro.2007.03.059

[mjz015C31] Shoelson S.E., Lee J., and Goldfine A.B. (2006). Inflammation and insulin resistance. J. Clin. Invest. 116, 1793–1801. 16823477 10.1172/JCI29069 PMC1483173

[mjz015C32] Simonet W.S., Lacey D.L., Dunstan C.R., et al. (1997). Osteoprotegerin: a novel secreted protein involved in the regulation of bone density. Cell 89, 309–319. 9108485 10.1016/s0092-8674(00)80209-3

[mjz015C33] Strissel K.J., Stancheva Z., Miyoshi H., et al. (2007). Adipocyte death, adipose tissue remodeling, and obesity complications. Diabetes 56, 2910–2918. 17848624 10.2337/db07-0767

[mjz015C34] Sun K., and Lai E.C. (2013). Adult-specific functions of animal microRNAs. Nat. Rev. Genet. 14, 535–548. 23817310 10.1038/nrg3471 PMC4136762

[mjz015C35] Takahashi K., Mizuarai S., Araki H., et al. (2003). Adiposity elevates plasma MCP-1 levels leading to the increased CD11b-positive monocytes in mice. J. Biol. Chem. 278, 46654–46660. 13129912 10.1074/jbc.M309895200

[mjz015C36] Tousoulis D., Siasos G., Maniatis K., et al. (2013). Serum osteoprotegerin and osteopontin levels are associated with arterial stiffness and the presence and severity of coronary artery disease. Int. J. Cardiol. 167, 1924–1928. 22640692 10.1016/j.ijcard.2012.05.001

[mjz015C37] Trajkovski M., Hausser J., Soutschek J., et al. (2011). MicroRNAs 103 and 107 regulate insulin sensitivity. Nature 474, 649–653. 21654750 10.1038/nature10112

[mjz015C38] Weisberg S.P., McCann D., Desai M., et al. (2003). Obesity is associated with macrophage accumulation in adipose tissue. J. Clin. Invest. 112, 1796–1808. 14679176 10.1172/JCI19246 PMC296995

[mjz015C39] White M.F. (2002). IRS proteins and the common path to diabetes. Am. J. Physiol. Endocrinol. Metab. 283, E413–E422. 12169433 10.1152/ajpendo.00514.2001

[mjz015C40] Xin M., Small E.M., Sutherland L.B., et al. (2009). MicroRNAs miR-143 and miR-145 modulate cytoskeletal dynamics and responsiveness of smooth muscle cells to injury. Genes Dev. 23, 2166–2178. 19720868 10.1101/gad.1842409 PMC2751981

[mjz015C41] Xu H., Barnes G.T., Yang Q., et al. (2003). Chronic inflammation in fat plays a crucial role in the development of obesity-related insulin resistance. J. Clin. Invest. 112, 1821–1830. 14679177 10.1172/JCI19451 PMC296998

[mjz015C42] Zhang M.L., Zheng B., Tong F., et al. (2017). iNOS-derived peroxynitrite mediates high glucose-induced inflammatory gene expression in vascular smooth muscle cells through promoting KLF5 expression and nitration. Biochim. Biophys. Acta 1863, 2821–2834. 10.1016/j.bbadis.2017.07.004 28711598

