# Supplementary material for: miR-145 improves metabolic inflammatory disease through multiple pathways
Source: J Mol Cell Biol. 2019 Apr 3;12(2):152–62. doi: 10.1093/jmcb/mjz015 (PMC7109608; doi:10.1093/jmcb/mjz015)
Supplement: mjz015_Supplementary_material [file mjz015_supplementary_material.pdf]

Supplementary Figures

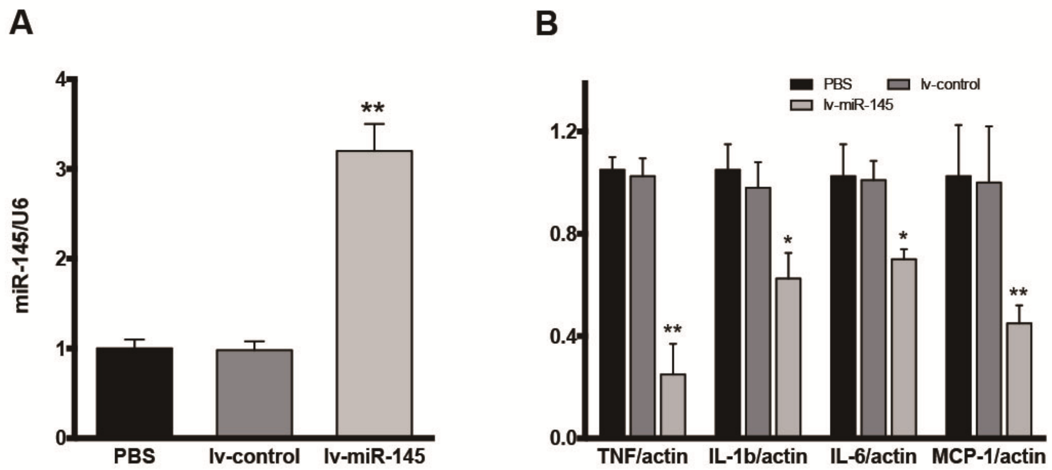

Supplementary fig 1. MiR-145 overexpression decreased proinflammatory cytokines in liver of db/db mice.

A, QPCR of miR-145 in liver of db/db mice treated with lv-miR-145, lv-control or PBS.

B, QPCR of MCP-1, TNF-α, IL-1β and IL-6 in liver of db/db mice treated with lv-miR-145, lv-control or PBS.

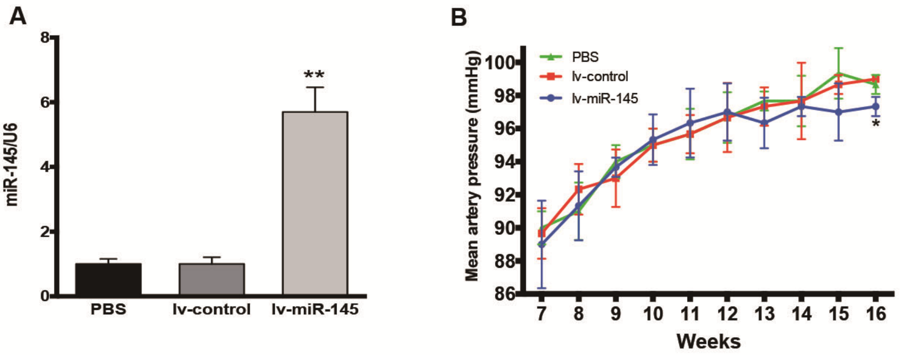

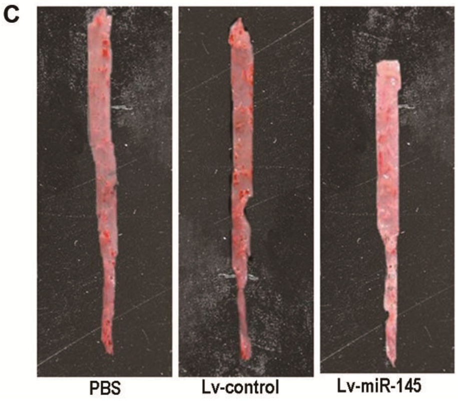

Supplementary fig 2. MiR-145 alleviate atherosclerosis in ApoE-/- mice.

A,QPCR of miR-145 in the aorta of ApoE-/- mice treated with lv-miR-145, lv-control or PBS.

B, Mean artery pressure of ApoE-/- mice after treated with lv-miR-145, lv-control or PBS.

C, Oil red staining of the en face aorta of ApoE-/- mice after treated with lv-miR-145, lv-control or PBS.

Supplementary Table S1 Relative expression profiles of miRNA microarrays

Name	Fold Change (Glucose/Control)	Fold Change (AcLDL/Control)	Fold Change (FFA/Control)	Fold Change (Glucose + AcLDL/Control)
hsa-let-7a	1.03	0.55	0.74	0.79
hsa-let-7b	1.2	0.52	0.81	0.86
hsa-let-7c	0.98	0.64	0.76	0.82
hsa-let-7d	1.06	0.52	0.74	0.79
hsa-let-7e	0.46	0.54	0.46	0.51
hsa-let-7f	0.92	0.5	0.66	0.72
hsa-let-7g	0.79	0.49	0.59	0.64
hsa-let-7i	0.9	0.49	0.65	0.7
hsa-miR-1	0.58	0.51	0.5	0.55
hsa-miR-100	0.76	0.59	0.63	0.68
hsa-miR-101	0.63	0.5	0.52	0.57
hsa-miR-103	0.4	0.55	0.44	0.48
hsa-miR-105	0.56	0.49	0.48	0.53
hsa-miR-106a	1.55	0.69	1.06	1.12
hsa-miR-106b	0.96	0.61	0.73	0.79
hsa-miR-107	0.61	0.58	0.55	0.6
hsa-miR-10a	0.57	0.52	0.5	0.55
hsa-miR-10b	0.8	0.67	0.69	0.74
hsa-miR-122a	0.62	0.61	0.57	0.62
hsa-miR-124a	0.59	0.62	0.56	0.61
hsa-miR-125a	0.58	0.69	0.6	0.65
hsa-miR-125b	0.72	0.62	0.62	0.68
hsa-miR-126	0.57	0.52	0.5	0.55
hsa-miR-126*	0.67	0.52	0.55	0.6
hsa-miR-127	0.39	0.49	0.4	0.44
hsa-miR-128a	0.38	0.48	0.39	0.44
hsa-miR-128b	0.77	0.57	0.62	0.67
hsa-miR-129	1	1.08	0.99	1.05
hsa-miR-130a	0.56	0.49	0.49	0.53
hsa-miR-130b	0.62	0.52	0.53	0.58
hsa-miR-132	0.84	0.75	0.74	0.8
hsa-miR-133a-133b	0.67	0.63	0.61	0.66
hsa-miR-133b	0.59	0.56	0.53	0.58
hsa-miR-134	0.57	0.52	0.51	0.55
hsa-miR-135a	0.69	0.64	0.62	0.67
hsa-miR-135b	0.58	0.57	0.53	0.58
hsa-miR-136	0.59	0.62	0.56	0.61
hsa-miR-137	0.57	0.53	0.51	0.55
hsa-miR-138	0.73	0.68	0.66	0.71
hsa-miR-139	0.67	0.58	0.58	0.63
hsa-miR-140	0.69	0.62	0.61	0.66

hsa-miR-141	0.61	0.6	0.56	0.61
hsa-miR-142-3p	1.27	0.48	0.82	0.87
hsa-miR-142-5p	0.58	0.5	0.5	0.54
hsa-miR-143	0.64	0.51	0.53	0.58
hsa-miR-144	0.62	0.58	0.56	0.61
**hsa-miR-145**	**0.49**	**0.48**	**0.44**	**0.48**
hsa-miR-146a	0.93	0.46	0.64	0.7
hsa-miR-146a	0.85	0.5	0.63	0.68
hsa-miR-146b	0.96	0.62	0.74	0.79
hsa-miR-147	0.44	0.71	0.54	0.59
hsa-miR-148a	0.7	0.56	0.59	0.64
hsa-miR-148b	0.41	0.53	0.43	0.48
hsa-miR-149	0.56	0.56	0.52	0.57
hsa-miR-150	0.73	0.61	0.63	0.68
hsa-miR-151	0.45	0.56	0.46	0.51
hsa-miR-152	0.59	0.53	0.51	0.56
hsa-miR-153	0.56	0.49	0.48	0.53
hsa-miR-154	0.71	0.62	0.62	0.67
hsa-miR-154*	0.66	0.61	0.59	0.64
hsa-miR-155	1.31	0.5	0.85	0.91
hsa-miR-15a	0.75	0.53	0.59	0.64
hsa-miR-15b	0.81	0.59	0.65	0.7
hsa-miR-16	1.19	0.46	0.77	0.83
hsa-miR-17-3p	0.49	0.49	0.45	0.5
hsa-miR-17-5p	1.28	0.6	0.88	0.94
hsa-miR-181a	0.67	0.68	0.63	0.68
hsa-miR-181b	0.68	0.59	0.59	0.64
hsa-miR-181c	0.56	0.57	0.52	0.57
hsa-miR-181d	0.58	0.55	0.52	0.57
hsa-miR-182	0.72	0.63	0.63	0.69
hsa-miR-182*	0.61	0.55	0.54	0.58
hsa-miR-183	0.6	0.59	0.55	0.6
hsa-miR-184	0.42	0.56	0.45	0.5
hsa-miR-185	0.76	0.55	0.61	0.66
hsa-miR-186	0.75	0.6	0.63	0.68
hsa-miR-187	0.64	0.56	0.56	0.61
hsa-miR-188	0.59	0.54	0.52	0.57
hsa-miR-189	0.59	0.5	0.5	0.55
hsa-miR-18a	0.96	0.51	0.68	0.74
hsa-miR-18a	1.15	0.63	0.83	0.89
hsa-miR-18a*	0.57	0.54	0.51	0.56
hsa-miR-18b	1.22	0.61	0.86	0.92
hsa-miR-191	1.05	0.45	0.7	0.75
hsa-miR-191*	0.38	0.66	0.48	0.53
hsa-miR-192	0.6	0.59	0.55	0.6
hsa-miR-193a	0.76	0.48	0.57	0.62

hsa-miR-193b	0.72	0.7	0.66	0.71
hsa-miR-194	0.73	0.62	0.63	0.68
hsa-miR-195	0.59	0.55	0.52	0.57
hsa-miR-195	0.56	0.51	0.49	0.54
hsa-miR-196a	0.57	0.52	0.5	0.55
hsa-miR-196b	0.63	0.65	0.6	0.65
hsa-miR-197	0.72	0.81	0.72	0.78
hsa-miR-198	0.6	0.67	0.59	0.64
hsa-miR-199a	0.61	0.52	0.52	0.57
hsa-miR-199a*	0.49	0.6	0.5	0.55
hsa-miR-199b	0.62	0.6	0.57	0.61
hsa-miR-19a	0.76	0.47	0.57	0.62
hsa-miR-19b	0.4	0.46	0.4	0.44
hsa-miR-200a	0.63	0.51	0.53	0.58
hsa-miR-200a*	0.58	0.58	0.54	0.59
hsa-miR-200b	0.59	0.54	0.52	0.57
hsa-miR-200c	0.59	0.5	0.5	0.55
hsa-miR-202	0.77	0.75	0.71	0.77
hsa-miR-202*	0.62	0.55	0.54	0.59
hsa-miR-203	0.63	0.56	0.55	0.6
hsa-miR-204	0.61	0.53	0.53	0.58
hsa-miR-205	0.41	0.54	0.44	0.48
hsa-miR-206	0.66	0.65	0.61	0.66
hsa-miR-208	0.61	0.51	0.52	0.56
hsa-miR-20a	1.43	0.68	0.99	1.06
hsa-miR-20a	1.37	0.59	0.92	0.98
hsa-miR-20b	0.69	0.56	0.58	0.63
hsa-miR-21	1.03	0.46	0.69	0.75
hsa-miR-210	0.54	0.53	0.49	0.54
hsa-miR-211	0.67	0.59	0.59	0.64
hsa-miR-212	0.99	1.41	1.15	1.22
hsa-miR-213	0.59	0.51	0.5	0.55
hsa-miR-214	0.42	0.5	0.42	0.47
hsa-miR-215	0.66	0.56	0.56	0.61
hsa-miR-216	0.43	0.55	0.45	0.49
hsa-miR-217	0.39	0.49	0.4	0.45
hsa-miR-218	0.54	0.51	0.48	0.53
hsa-miR-219	0.46	0.68	0.53	0.58
hsa-miR-22	0.75	0.58	0.62	0.67
hsa-miR-220	0.41	0.54	0.44	0.48
hsa-miR-221	0.73	0.58	0.61	0.66
hsa-miR-222	0.92	0.68	0.75	0.81
hsa-miR-223	0.84	0.53	0.64	0.69
hsa-miR-224	0.31	0.54	0.39	0.43
hsa-miR-23a	0.61	0.64	0.58	0.63
hsa-miR-23b	0.7	0.74	0.68	0.73

hsa-miR-24	1.04	0.6	0.77	0.83
hsa-miR-25	0.58	0.57	0.53	0.58
hsa-miR-26b	0.76	0.59	0.63	0.68
hsa-miR-27a	0.78	0.61	0.65	0.7
hsa-miR-27b	0.79	0.71	0.71	0.76
hsa-miR-28	0.6	0.55	0.53	0.58
hsa-miR-296	0.77	0.64	0.66	0.71
hsa-miR-299-3p	0.69	0.79	0.7	0.75
hsa-miR-299-5p	0.66	0.65	0.61	0.66
hsa-miR-29a	1.24	0.47	0.8	0.86
hsa-miR-29b	0.64	0.49	0.52	0.57
hsa-miR-29c	0.84	0.62	0.68	0.73
hsa-miR-302a	0.69	0.63	0.61	0.67
hsa-miR-302a*	0.48	0.55	0.47	0.52
hsa-miR-302b	0.57	0.52	0.5	0.55
hsa-miR-302b*	0.69	0.56	0.58	0.63
hsa-miR-302c	0.58	0.49	0.49	0.54
hsa-miR-302c*	0.67	0.68	0.63	0.68
hsa-miR-302d	0.63	0.52	0.54	0.58
hsa-miR-30a-3p	0.57	0.5	0.49	0.54
hsa-miR-30a-5p	1.03	0.57	0.74	0.8
hsa-miR-30b	0.84	0.64	0.69	0.75
hsa-miR-30c	0.95	0.46	0.65	0.71
hsa-miR-30d	1.04	1.63	1.28	1.36
hsa-miR-30e-3p	0.67	0.53	0.56	0.61
hsa-miR-30e-5p	1.07	0.64	0.8	0.86
hsa-miR-31	0.43	0.48	0.42	0.46
hsa-miR-32	0.88	0.61	0.69	0.75
hsa-miR-320	1.02	0.99	0.95	1.01
hsa-miR-323	0.35	0.56	0.42	0.47
hsa-miR-324-3p	0.44	0.69	0.52	0.57
hsa-miR-324-5p	0.75	1.32	0.99	1.06
hsa-miR-325	0.64	0.59	0.57	0.62
hsa-miR-326	0.63	0.6	0.57	0.62
hsa-miR-328	0.61	0.53	0.53	0.58
hsa-miR-33	0.78	0.55	0.62	0.67
hsa-miR-330	0.6	0.52	0.52	0.56
hsa-miR-331	0.58	0.51	0.5	0.55
hsa-miR-335	0.69	0.57	0.59	0.64
hsa-miR-337	0.69	0.53	0.57	0.61
hsa-miR-338	0.63	0.52	0.53	0.58
hsa-miR-339	0.59	0.5	0.5	0.55
hsa-miR-340	0.42	0.55	0.45	0.49
hsa-miR-342	0.78	0.64	0.66	0.72
hsa-miR-345	0.68	0.55	0.57	0.62
hsa-miR-346	0.75	0.98	0.82	0.88

hsa-miR-34a	0.6	0.56	0.53	0.58
hsa-miR-34b	0.69	0.6	0.6	0.65
hsa-miR-34c	0.67	0.58	0.58	0.63
hsa-miR-362	0.6	0.5	0.5	0.55
hsa-miR-363	0.73	0.58	0.61	0.66
hsa-miR-365	0.69	0.57	0.59	0.64
hsa-miR-367	0.57	0.54	0.51	0.56
hsa-miR-368	0.57	0.48	0.48	0.53
hsa-miR-369-3p	0.67	0.61	0.6	0.65
hsa-miR-369-5p	0.67	0.63	0.61	0.66
hsa-miR-370	0.66	0.63	0.6	0.65
hsa-miR-371	0.58	0.58	0.54	0.59
hsa-miR-372	0.58	0.5	0.5	0.54
hsa-miR-373	0.71	0.64	0.63	0.68
hsa-miR-373*	1.25	1.67	1.4	1.48
hsa-miR-374	0.58	0.51	0.5	0.55
hsa-miR-375	0.61	0.82	0.67	0.73
hsa-miR-376a	0.66	0.61	0.59	0.64
hsa-miR-376b	0.44	0.53	0.45	0.49
hsa-miR-377	0.61	0.49	0.5	0.55
hsa-miR-378	0.57	0.53	0.51	0.56
hsa-miR-379	0.69	0.62	0.61	0.66
hsa-miR-380-3p	0.42	0.55	0.45	0.49
hsa-miR-380-5p	0.57	0.53	0.51	0.55
hsa-miR-381	0.62	0.54	0.54	0.58
hsa-miR-382	0.59	0.54	0.52	0.57
hsa-miR-383	0.67	0.62	0.6	0.65
hsa-miR-384	0.62	0.51	0.52	0.57
hsa-miR-409-3p	0.59	0.64	0.57	0.62
hsa-miR-409-3p	0.61	0.55	0.54	0.58
hsa-miR-409-5p	0.58	0.5	0.5	0.55
hsa-miR-410	0.64	0.66	0.6	0.65
hsa-miR-412	0.41	0.6	0.47	0.51
hsa-miR-422a	0.6	0.58	0.55	0.6
hsa-miR-422b	0.64	0.57	0.56	0.61
hsa-miR-423	0.91	0.79	0.8	0.86
hsa-miR-424	0.67	0.52	0.55	0.6
hsa-miR-425	0.58	0.53	0.51	0.56
hsa-miR-429	0.68	0.59	0.59	0.64
hsa-miR-431	0.58	0.51	0.51	0.55
hsa-miR-432	0.69	0.66	0.63	0.68
hsa-miR-432*	0.57	0.66	0.57	0.62
hsa-miR-433	0.69	0.58	0.59	0.64
hsa-miR-448	0.65	0.53	0.55	0.6
hsa-miR-449	0.59	0.52	0.51	0.56
hsa-miR-450	0.55	0.5	0.49	0.53

hsa-miR-451	0.66	0.64	0.6	0.65
hsa-miR-451	0.65	0.57	0.57	0.62
hsa-miR-452	0.64	0.67	0.62	0.67
hsa-miR-452*	0.58	0.62	0.56	0.6
hsa-miR-453	0.58	0.58	0.54	0.58
hsa-miR-455	0.66	0.63	0.6	0.65
hsa-miR-483	0.7	0.63	0.62	0.67
hsa-miR-484	0.67	0.6	0.59	0.64
hsa-miR-485-3p	0.58	0.56	0.53	0.57
hsa-miR-485-5p	0.67	0.79	0.69	0.74
hsa-miR-486	0.56	0.55	0.51	0.56
hsa-miR-487a	0.72	0.65	0.64	0.69
hsa-miR-489	0.59	0.57	0.54	0.58
hsa-miR-490	0.42	0.52	0.43	0.48
hsa-miR-491	0.85	0.61	0.68	0.74
hsa-miR-492	0.77	0.63	0.65	0.7
hsa-miR-493-5p	0.61	0.48	0.5	0.55
hsa-miR-494	1.01	0.95	0.93	0.99
hsa-miR-495	0.77	0.58	0.63	0.68
hsa-miR-496	0.61	0.5	0.51	0.56
hsa-miR-497	0.6	0.49	0.5	0.55
hsa-miR-498	1.1	2.02	1.51	1.59
hsa-miR-499	0.69	0.64	0.62	0.67
hsa-miR-500	0.68	0.57	0.58	0.63
hsa-miR-501	0.59	0.61	0.56	0.61
hsa-miR-502	0.59	0.52	0.51	0.56
hsa-miR-503	0.93	0.79	0.81	0.86
hsa-miR-504	0.62	0.57	0.55	0.6
hsa-miR-505	0.44	0.62	0.49	0.54
hsa-miR-506	0.62	0.5	0.52	0.57
hsa-miR-507	0.75	0.62	0.64	0.69
hsa-miR-508	0.66	0.56	0.57	0.62
hsa-miR-509	0.59	0.52	0.51	0.56
hsa-miR-510	0.57	0.5	0.49	0.54
hsa-miR-511	0.74	0.62	0.64	0.69
hsa-miR-512-3p	0.67	0.51	0.55	0.6
hsa-miR-512-5p	0.57	0.57	0.52	0.57
hsa-miR-513	0.41	0.54	0.43	0.48
hsa-miR-514	0.7	0.58	0.59	0.64
hsa-miR-515-3p	0.65	0.52	0.54	0.59
hsa-miR-515-5p	0.59	0.47	0.49	0.54
hsa-miR-516-3p	0.58	0.49	0.49	0.54
hsa-miR-516-5p	0.46	0.63	0.51	0.55
hsa-miR-517*	0.7	0.67	0.64	0.69
hsa-miR-517a	0.68	0.53	0.56	0.61
hsa-miR-517a-517b	0.57	0.58	0.53	0.58

hsa-miR-517c	0.56	0.52	0.5	0.55
hsa-miR-518a	0.56	0.65	0.56	0.61
hsa-miR-518b	0.66	0.58	0.58	0.63
hsa-miR-518c	0.58	0.52	0.51	0.56
hsa-miR-518c*	0.68	0.59	0.59	0.64
hsa-miR-518d	0.43	0.46	0.41	0.45
hsa-miR-518e	0.65	0.59	0.58	0.63
hsa-miR-518f	0.63	0.53	0.54	0.58
hsa-miR-518f*-526a	0.57	0.47	0.48	0.53
hsa-miR-519a	0.58	0.48	0.49	0.53
hsa-miR-519b	0.58	0.47	0.49	0.53
hsa-miR-519c	0.56	0.51	0.49	0.54
hsa-miR-519d	0.76	0.9	0.78	0.84
hsa-miR-519e	0.66	0.75	0.66	0.71
hsa-miR-519e*	0.41	0.53	0.43	0.48
hsa-miR-520a	0.58	0.53	0.51	0.56
hsa-miR-520a*	0.59	0.56	0.54	0.58
hsa-miR-520b-520c	0.58	0.47	0.48	0.53
hsa-miR-520d	0.47	0.57	0.48	0.52
hsa-miR-520d*	0.73	0.64	0.64	0.69
hsa-miR-520e	0.6	0.57	0.54	0.59
hsa-miR-520f-520c	0.42	0.66	0.5	0.55
hsa-miR-520g	0.55	0.48	0.47	0.52
hsa-miR-520g-520h	0.57	0.55	0.52	0.57
hsa-miR-521	0.68	0.61	0.6	0.65
hsa-miR-522	0.59	0.6	0.56	0.61
hsa-miR-523	0.59	0.53	0.52	0.57
hsa-miR-524*	0.71	0.61	0.61	0.66
hsa-miR-525	0.68	0.61	0.6	0.65
hsa-miR-525*-524	0.58	0.53	0.51	0.56
hsa-miR-526b	0.65	0.52	0.54	0.59
hsa-miR-526b*	0.64	0.62	0.59	0.64
hsa-miR-526c	0.58	0.59	0.55	0.59
hsa-miR-527-518a-2*	0.6	0.48	0.49	0.54
hsa-miR-7	0.61	0.49	0.5	0.55
hsa-miR-7	0.58	0.68	0.59	0.64
hsa-miR-9	0.59	0.52	0.51	0.56
hsa-miR-9*	0.6	0.56	0.54	0.59
hsa-miR-92	0.93	0.82	0.82	0.88
hsa-miR-93	0.62	0.51	0.52	0.57
hsa-miR-95	0.61	0.5	0.51	0.56
hsa-miR-96	0.59	0.5	0.51	0.55
hsa-miR-98	0.59	0.52	0.51	0.56
hsa-miR-99a	0.69	0.63	0.62	0.67
hsa-miR-99b	0.69	0.55	0.58	0.63

Supplementary Table S2 Expression profiles of inflammatory cytokines in liver of C57BL/6J mice treated with control, miR-145 ASO, or miR-145.

Cytokines & Chemokines(pg/ml)	Control	miR-145 ASO	miR-145
TNF-α	345.44 ± 50.72	568.92 ± 124.22*	417.28 ± 94.36
IL-1α	230.64 ± 76.45	198.60 ± 20.02	213.69 ± 57.64
IL-6	1721.14 ± 170.33	1561.96 ± 148.74	1584.49 ± 270.62
IL-8	377.60 ± 37.18	396.36 ± 35.55	372.56 ± 27.744
IL-10	526.15 ± 64.71	754.94 ± 60.36*	579.34 ± 145.57
MCP-1	237.13 ± 66.22	348.43 ± 48.86*	277.17 ±44.84
CRP	258.46 ± 85.34	305.69 ± 37.61	267.69 ± 27.47

TNF-α, tumor necrosis factor-α; IL-1α, interleukin-1α; IL-6, interleukin-6; IL-8, interleukin-8; IL-10, interleukin-10; MCP-1, monocyte chemotactic protein-1; CRP, C-reactionprotein. Data are mean ± SD (*P <0 05 vs. control).

Supplementary Table S3 Quantitative assessment of atherosclerosis in ApoE $^{-/-}$ mice.

Group	Plaque area(um^2)	Lumen area(um^2)	Plaque area/ Lumen area(%)
PBS	3603	17162	21
lv-miR-control	5889	21730	27
lv-miR-145	2565	27997	9**

** P<0.01
